# Noise Enhanced Signal Detection of Variable Detectors under Certain Constraints

**DOI:** 10.3390/e20060470

**Published:** 2018-06-17

**Authors:** Ting Yang, Shujun Liu, Wenguo Liu, Jishun Guo, Pin Wang

**Affiliations:** 1 College of Communication Engineering, Chongqing University, Chongqing 400044, China; 2 Chongqing Institution of Tuberculosis Treatment and Prevention, Chongqing 400050, China; 3 GAC Automotive Engineering Institute, Guangzhou 510640, China

**Keywords:** noise enhanced, hypothesis-testing, Bayes risk, variable detector

## Abstract

In this paper, a noise enhanced binary hypothesis-testing problem was studied for a variable detector under certain constraints in which the detection probability can be increased and the false-alarm probability can be decreased simultaneously. According to the constraints, three alternative cases are proposed, the first two cases concerned minimization of the false-alarm probability and maximization of the detection probability without deterioration of one by the other, respectively, and the third case was achieved by a randomization of two optimal noise enhanced solutions obtained in the first two limit cases. Furthermore, the noise enhanced solutions that satisfy the three cases were determined whether randomization between different detectors was allowed or not. In addition, the practicality of the third case was proven from the perspective of Bayes risk. Finally, numerous examples and conclusions are presented.

## 1. Introduction

Stochastic resonance (SR) is a physical phenomenon where noise plays an active role in enhancing the performance of some nonlinear systems under certain conditions. Since the concept of SR was first put forward by Benzi et al. in 1981 [1], the positive effect of SR has been widely investigated and applied in various research fields, such as physics, chemical, biological and electronic, etc. [2,3,4,5,6,7,8,9,10]. In signal detection theory, it is also called noise enhanced detection [3]. The classical signature of noise enhanced detection can be an increase in output signal-to-noise ratio (SNR) [11,12,13] or mutual information (MI) [14,15,16,17,18], a decrease in Bayes risk [19,20,21] or probability of error [22], or an increase in detection probability without increasing the false-alarm probability [23,24,25,26,27,28].

Studies in recent years indicate that the detection performance of a nonlinear detector in a hypothesis testing problem can be improved by adding additive noise to the system input or adjusting the background noise level based on the Bayesian [19], Minimax [20] or Neyman–Pearson [24,25,28] criteria. In [19], S. Bayram et al. analyzed the noise enhanced M-ary composite hypothesis testing problem in a restricted Bayesian framework. Specifically, the minimax criterion can be used when the prior probabilities are unknown. The research results showed that the noise enhanced detection in the Minimax framework [20] can be viewed as a special case in the restricted Bayesian framework.

Numerous researches on how to increase the detection probability according to the Neyman–Pearson criterion have been made. In [23], S. Kay showed that for the detection of a direct current (DC) signal in a Gaussian mixture noise background, the detection probability of a sign detector can be enhanced by adding a suitable white Gaussian noise under certain conditions. A mathematical framework was established by H. Chen et al. in order to analyze the mechanism of the SR effect on the binary hypothesis testing problem according to Neyman–Pearson criterion for a fixed detector in [24]. The optimal additive noise that maximizes the detection probability without decreasing the false-alarm probability and its probability density function (pdf) are derived in detail. In addition, the conditions sufficing for improvability and non-improvability are given. In [27], Ashok Patel and Bart Kosko presented theorems and an algorithm to search the optimal or near-optimal additive noise for the same problem as in [24] from another perspective. In [28], binary noise enhanced composite hypothesis-testing problems are investigated according to the Max-sum, Max-min and Max-max criteria, respectively. Furthermore, a noise enhanced detection problem for a variable detector according to Neyman–Pearson criterion is investigated in [25]. Similar to [24,27,28], it also only considers how to increase the detection probability but ignores the importance of decreasing the false-alarm probability.

Few researchers focus on how to reduce the false-alarm probability and there is no evidence to indicate that the false-alarm probability cannot be decreased by adding additive noise on the premise of not deteriorating the detection probability. In fact, it is significant to decrease the false-alarm probability without decreasing the detection probability. In [2], a noise enhanced model for a fixed detector is proposed, which considers how to use the additive noise to decrease the false-alarm probability and increase the detection probability simultaneously. However, unfortunately, it does not take into account the case where the detector is variable. When no randomization exists between different detectors, we just need to find the most appropriate detector since the optimum solution for each detector can be obtained straightforwardly by utilizing the results in [2]. On the other hand, if the randomization between different detectors is allowed, some new noise enhanced solutions can be introduced by the randomization of multiple detector and additive noise pairs. Therefore, the aim of this paper was to find the optimal noise enhanced solutions for the randomization case.

Actually, in many cases, although the structure of the detector cannot be altered, some of its parameters can be adjusted to obtain a better performance. Even in some particular situations, the structure can also be changed. In this paper, we consider the noise enhanced model established in [2] for a variable detector, where a candidate set of decision functions can be utilized. Instead of solving the model directly, three alternative cases are considered. The first two cases are to minimize the false-alarm probability and maximize the detection probability without deterioration of one by the other, respectively. When the randomization between the detectors cannot be allowed, the first two cases can be realized by choosing a suitable detector and adding the corresponding optimal additive noise. When the randomization between different detectors can be allowed, the optimal noise enhanced solutions for the first two cases are suitable randomization between the two detectors and additive noise pairs. Whether the randomization between the detectors is allowed or not, the last case can be obtained by a convex combination of the optimal noise enhanced solutions for the first two cases with corresponding weights. In addition, the noise enhanced model also provides a solution to reduce the Bayes risk for the variable detector in this paper, which is different from the minimization of Bayes risk under Bayesian criterion in [19] where the false-alarm and detection probabilities are not of concern.

The remainder of this paper is organized as follows. In Section 2, a noise enhanced binary hypothesis-testing model for a variable detector is established, which is simplified into three different cases. In Section 3, the forms of the noise enhanced solutions are discussed. Furthermore, the exact parameter values of these noise enhanced solutions are determined in Section 4 allowing randomization between the detectors. Numerous results are presented in Section 5 and the conclusions are provided in Section 6.

Notation: Lower-case bold letters denote vectors, x is a K-dimensional observation vector; upper-case hollow letters denote sets, e.g., ℝ denotes a set of real numbers; p(⋅) is used to denote pdf, while p(⋅|⋅) its corresponding conditional counterpart; ϕ denotes the decision function, Φ denotes a set of decision functions; δ(⋅) denotes the Dirac function; ∗, ∑, ∫, E{⋅}, min, max and arg denote convolution, summation, integral, expectation, minimum, maximum and argument operators, respectively; inf{⋅} and sup{⋅} denote infimum and supremum operators, respectively; $\varphi (\cdot ;\mu ,\sigma^{2})$ denotes a Gaussian pdf with mean μ and variance $\sigma^{2}$.

## 2. Noise Enhanced Detection Model for Binary Hypothesis-Testing

### 2.1. Problem Formulation

A binary hypothesis-testing problem is considered as follows

(1) $$H_{i}:p_{i}(x),i=0,1,$$

where $H_{0}$ and $H_{1}$ denote the original and alternative hypotheses, respectively, x is a K-dimensional observation vector, i.e., $x\in {\mathbb{R}}^{K}$, ℝ denotes a set of real numbers, and $p_{i}(x)$ is the pdf of x under $H_{i}$, i=0,1. Let ϕ(x) represent the decision function, which is also the probability of choosing $H_{1}$, and 0≤ϕ(x)≤1. For a given ϕ, the original false-alarm probability $P_{FA}^{x}$ and detection probability $P_{D}^{x}$ can be calculated as

(2) $$P_{FA}^{x}=\int_{{\mathbb{R}}^{K}}\phi (x)p_{0}(x)dx,$$

(3) $$P_{D}^{x}=\int_{{\mathbb{R}}^{K}}\phi (x)p_{1}(x)dx.$$

The new noise modified observation y is obtained by adding an independent additive noise n to the original observation x such that

(4) y=x+n.

Then the pdf of y under $H_{i}$ can be formulated as the following convolutions of $p_{i}(\cdot )$ and $p_{n}(\cdot )$,

(5) $$p_{y}(y|H_{i})=p_{n}(\cdot )*p_{i}(\cdot )=\int_{{\mathbb{R}}^{K}}p_{n}(n)p_{i}(y-n)dn.$$

The noise modified false-alarm probability $P_{FA,\phi }^{y}$ and detection probability $P_{D,\phi }^{y}$ for the given ϕ can be calculated by

(6) $$\begin{array}{cc} P_{FA,\phi }^{y} & =\int_{{\mathbb{R}}^{K}}\phi (y)p_{y}(y|H_{0})dy=\int_{{\mathbb{R}}^{K}}p_{n}(n)\left(\int_{{\mathbb{R}}^{K}}\phi (y)p_{0}(y-n)dy\right)dn\\ & =\int_{{\mathbb{R}}^{K}}p_{n}(n)F_{0,\phi }(n)dn=E_{n}(F_{0,\phi }(n)) \end{array},$$

(7) $$P_{D,\phi }^{y}=\int_{{\mathbb{R}}^{K}}\phi (y)p_{y}(y|H_{1})dy=\int_{{\mathbb{R}}^{K}}p_{n}(n)F_{1,\phi }(n)dn=E_{n}(F_{1,\phi }(n)),$$

such that

(8) $$F_{i,\phi }(n)=\int_{{\mathbb{R}}^{K}}\phi (y)p_{i}(y-n)dy,\;\;i=0,1.$$

From (6) and (7), $P_{FA,\phi }^{y}$ and $P_{D,\phi }^{y}$ are the respective expected values of $F_{0,\phi }(n)$ and $F_{1,\phi }(n)$ based on the distribution of the additive noise $p_{n}(\cdot )$. Especially, $P_{FA}^{x}=F_{0,\phi }(0)$ and $P_{D}^{x}=F_{1,\phi }(0)$ for the given ϕ according to (8).

### 2.2. Noise Enhanced Detection Model for a Variable Detector

Actually, although the detector cannot be substituted in many cases, some parameters of the detectors can be adjusted to achieve a better detection performance, such as the decision threshold. Even in some particular cases, the structure of the detector can also be altered. Instead of the strictly fixed ϕ(⋅), a candidate set of decision functions Φ is provided to be utilized here. As a result, for a variable detector, the optimization of detection performance can be achieved by adding a suitable noise and/or changing the detector. If the randomization between the detectors is allowed, the optimal solution of the noise enhanced detection problem would be a combination of multiple decision function and additive noise pairs.

Under the constraints that $P_{FA}^{y}\leq P_{FA}^{x}$ and $P_{D}^{y}\geq P_{D}^{x}$, a noise enhanced detection model for a variable detector is established as follows

(9) $$\left\{ \begin{array}{ccc} P_{FA}^{y}=P_{FA}^{x}-z_{1} & , & 0\leq z_{1}\leq P_{FA}^{x}\\ P_{D}^{y}=P_{D}^{x}+z_{2} & , & 0\leq z_{2}\leq 1-P_{D}^{x} \end{array}\right.,$$

where $z_{1}$ and $z_{2}$ represent the improvements of false-alarm and detection probabilities, respectively. Let z be the overall improvement of the detectability. Namely, z is the sum of $z_{1}$ and $z_{2}$ such that $z=z_{1}+z_{2}$.

It is obvious that the ranges of $z_{1}$ and $z_{2}$ are limited and the maximum values of $z_{1}$ and $z_{2}$ cannot be obtained at the same time. In order to solve the noise enhanced detection model, we can first consider two limit cases, i.e., the noise enhanced optimization problems of maximizing $z_{1}$ and $z_{2}$, respectively, under the constraints that $P_{FA}^{y}\leq P_{FA}^{x}$ and $P_{D}^{y}\geq P_{D}^{x}$,. Then a new suitable solution of the noise enhanced detection model in (9) can be obtained by a convex combination of two optimal noise enhanced solutions obtained in the two limit cases with corresponding weights. Consequently, the new noise enhanced solution can always guarantee $P_{FA}^{y}\leq P_{FA}^{x}$ and $P_{D}^{y}\geq P_{D}^{x}$, and the corresponding value of z is between the values of z obtained in the two limit cases. The three cases discussed above can be formulated as below.

(i) When $P_{D}^{y}\geq P_{D}^{x}$, the minimization of $P_{FA}^{y}$ is explored such that the maximum achievable $z_{1}$ is denoted by $z_{1}^{o}$, the corresponding $z_{2}$ is remarked as $z_{2}^{\left(i\right)}$ and $z_{2}^{\left(i\right)}\geq 0$. Thus, the corresponding false-alarm and detection probabilities can be written as below,
  (10) $$\left\{ \begin{array}{l} P_{FA,opt}^{y}=P_{FA}^{x}-z_{1}^{o}\\ P_{D}^{y}=P_{D}^{x}+z_{2}^{\left(i\right)} \end{array}\right..$$
(ii) When $P_{FA}^{y}\leq P_{FA}^{x}$, the maximum $P_{D}^{y}$ is searched such that the corresponding $z_{1}$ and $z_{2}$ are denoted by $z_{1}^{\left(ii\right)}$ and $z_{2}^{o}$, respectively, where $z_{1}^{\left(ii\right)}\geq 0$ and $z_{2}^{o}$ is the maximum achievable $z_{2}$. The corresponding false-alarm and detection probabilities can be expressed by
  (11) $$\left\{ \begin{array}{l} P_{FA}^{y}=P_{FA}^{x}-z_{1}^{\left(ii\right)}\\ P_{D,opt}^{y}=P_{D}^{x}+z_{2}^{o} \end{array}\right..$$
(iii) A noise enhanced solution obtained as a randomization between two optimal solutions of case (i) and case (ii) with weights η and 1−η, respectively, is applied in this case. Combining (10) and (11), the corresponding false-alarm and detection probabilities are calculated by
  (12) $$\left\{ \begin{array}{l} P_{FA}^{y}=P_{FA}^{x}-\left[\eta z_{1}^{o}+(1-\eta )z_{1}^{\left(ii\right)}\right]<P_{FA}^{x}\\ P_{D}^{y}=P_{D}^{x}+\left[\eta z_{2}^{\left(i\right)}+(1-\eta )z_{2}^{o}\right]>P_{D}^{x} \end{array}\right..$$

Naturally, $z_{1}=\eta z_{1}^{o}+(1-\eta )z_{1}^{\left(ii\right)}$ and $z_{2}=\eta z_{2}^{\left(i\right)}+(1-\eta )z_{2}^{o}$. It is obvious that case (iii) is identical to cases (i) when η=1, while case (iii) is the same as case (ii) when η=0. In addition, when η∈(0,1), more different noise enhanced solutions can be obtained by adjusting the value of η to increase the detection probability and decrease the false-alarm probabilities simultaneously.

Remarkably, if $P_{FA}^{y}<\alpha$ and $P_{D}^{y}>\beta$ are required, we only need to replace $P_{FA}^{x}$ and $P_{D}^{x}$ in this model with α and β, respectively.

## 3. Form of Noise Enhanced Solution under Different Situations

For the noise enhanced detection problem, when the detector is fixed, we only need to consider how to find the suitable or optimal additive noise. Nevertheless, when the detector is variable, we also need to consider how to choose a suitable detector. In this section, the noise enhanced detection problem for the variable detector is discussed for the case where the randomization between different detectors is allowed or not.

### 3.1. No Randomization between Detectors

For the case where no randomization between detectors is allowed, only one detector can be applied for each decision, thereby the noise enhanced detection problem for the variable detector can be simplified to that for a fixed detector $\phi_{o}$. That means the optimal noise enhanced solution is to find the optimal detector $\phi_{o}$ from Φ and add the corresponding optimal additive noise. The actual noise modified false-alarm and detection probabilities can be expressed by $P_{FA}^{y}=P_{FA,\phi_{o}}^{y}$ and $P_{D}^{y}=P_{D,\phi_{o}}^{y}$.

For any ϕ∈Φ, the corresponding $z_{1,\phi }^{o}$ and $z_{2,\phi }^{o}$ can be obtained straightforwardly by utilizing the results in [2]. Then the optimal detectors corresponding to case (i) and case (ii) can be selected as $\phi_{i}=\arg\underset{\phi \in \Phi }{\max}z_{1,\phi }^{o}$ and $\phi_{ii}=\arg\underset{\phi \in \Phi }{\max}z_{2,\phi }^{o}$. When $\phi_{i}=\phi_{ii}$, the optimal detector for case (iii) is selected as $\phi_{i}$. When $\phi_{i}\neq \phi_{ii}$, if $z_{\phi_{i}}=z_{1,\phi_{i}}^{o}+z_{2,,\phi_{i}}^{\left(i\right)}>z_{\phi_{ii}}=z_{1,,\phi_{ii}}^{\left(ii\right)}+z_{2,\phi_{ii}}^{o}$, $\phi_{i}$ is selected as the optimal detector for case (iii). Otherwise, $\phi_{ii}$ is selected.

### 3.2. Randomization between Detectors

For the case where the randomization between different detectors is allowed, multiple detector and additive noise pairs can be utilized for each decision, thereby the actual noise modified false-alarm and detection probabilities can be expressed as

(13) $$\left\{ \begin{array}{l} P_{FA}^{y}=\sum_{i=1}^{L}\xi_{i}P_{FA,\phi_{i}}^{y}\\ P_{D}^{y}=\sum_{i=1}^{L}\xi_{i}P_{D,\phi_{i}}^{y} \end{array}\right.,$$

where L≥1 is the number of detectors involved in the noise enhanced solution, $P_{FA,\phi_{i}}^{x}$ and $P_{D,\phi_{i}}^{x}$ are the respective false-alarm and detection probabilities for $\phi_{i}\in \Phi$, $\xi_{i}$ is the probability of $\phi_{i}$, and $0\leq \xi_{i}\leq 1$.

Let $f_{1}=F_{1,\phi }(n)$, then we have $n=F_{1,\phi }^{-1}(f_{1})$ and $f_{0}=F_{0,\phi }(n)=F_{0,\phi }(F_{1,\phi }^{-1}(f_{1}))$ where $F_{1,\phi }^{-1}$ is a function which maps $f_{1}$ to n based on function $F_{1,\phi }$. Thus $f_{0}$ can be a one-to-one or one-to-multiple function with respect to (w.r.t.) $f_{1}$, and vice versa. In addition, let U be the set of all pairs of $\left(f_{0},f_{1}\right)$, i.e., $U=\{(f_{0},f_{1})|f_{0}=F_{0,\phi }(n),f_{1}=F_{1,\phi }(n),n\in {\mathbb{R}}^{K},\phi \in \Phi \}$. On the basis of these definitions, the forms of the optimal enhanced solutions for case (i) and case (ii) can be presented in the following theorem.

**Theorem** **1.** *The optimal noise enhanced solution for case (i) (case (ii)) is a randomization of at most two detectors and discrete vector pairs, i.e.,* $\left[\phi_{1},n_{1}\right]$ *and* $\left[\phi_{2},n_{2}\right]$ *with the corresponding probabilities. The corresponding proof is presented in Appendix A and omitted here.*

## 4. Solutions of the Noise Enhanced Model with Randomization

In this section, we will explore and find the optimal enhanced solutions corresponding to cases (i) and (ii), then achieve case (iii) through utilizing the solutions of cases (i) and (ii). From (8), $f_{0}=F_{0,\phi }(n)$ and $f_{1}=F_{1,\phi }(n)$ can be treated as the false-alarm and detection probabilities, respectively, which are obtained by choosing a suitable ϕ∈Φ and adding a discrete vector n to x. Thus we can find the minimum $f_{0}$ marked as $F_{0m}$ and the maximum $f_{1}$ denoted by $F_{1M}$ from the set U. Then realizations of the case (i) and case (ii) can start with $F_{0m}$ and $F_{1M}$, respectively. A more detailed solving process is given as follows.

### 4.1. The Optimal Noise Enhanced Solution for Case (i)

In this subsection, the main goal is to determine the exact values of two detectors and constant vector pairs in the optimal noise enhanced solution for case (i).

Define $\Gamma_{\phi }(f_{1})=\inf(f_{0}:F_{1,\phi }(n)=f_{1})$ and $\Gamma (f_{1})=\underset{\phi \in \Phi }{\inf}(\Gamma_{\phi }(f_{1}))$. Namely, $\Gamma_{\phi }(f_{1})$ and $\Gamma (f_{1})$ are the minimum $f_{0}$ corresponding to a given $f_{1}$ for a fixed ϕ and for all ϕ∈Φ, respectively. According to the definitions of $F_{0m}$ and $\Gamma (f_{1})$, $F_{0m}$ is rewritten as $F_{0m}=\min(\Gamma (f_{1}))$, and the maximum $f_{1}$ corresponding to $F_{0m}$ can be denoted by $f_{1}^{*}=\arg\underset{f_{1}}{\max}(\Gamma (f_{1})=F_{0m})$. Combining with the location of $f_{1}^{*}$ and Theorem 1, we have the following theorem.

**Theorem** **2.** If $f_{1}^{*}\geq P_{D}^{x}$, then $P_{FA,opt}^{y}=F_{0m}<P_{FA}^{y}$ and $P_{D}^{y}=f_{1}^{*}\geq P_{D}^{x}$, the minimum achievable $P_{FA}^{y}$ is obtained by choosing the detector $\phi_{1o}$ and adding a discrete vector $n_{1o}$. If $f_{1}^{*}<P_{D}^{x}$, the optimal noise enhanced solution that minimizes $P_{FA}^{y}$ is a randomization of two detectors and discrete vector pairs, i.e., $\left[\phi_{11},n_{11}\right]$ and $\left[\phi_{12},n_{12}\right]$ with probabilities ζ and 1−ζ, and the corresponding $P_{D}^{y}=P_{D}^{x}$. The corresponding proof is given in Appendix B.

Obviously, when $f_{1}^{*}\geq P_{D}^{x}$, the detector $\phi_{1o}$ and constant vector $n_{1o}$ that minimizes $P_{FA}^{y}$ can be determined by $F_{0,\phi_{1o}}(n_{1o})=F_{0m}$ and $F_{1,\phi_{1o}}(n_{1o})=f_{1}^{*}$. Moreover, $z_{1}^{o}=P_{FA}^{x}-F_{0m}$ and $z_{2}^{\left(i\right)}=f_{1}^{*}-P_{D}^{x}$.

In order to determine the exact values of $\left[\phi_{11},n_{11}\right]$, $\left[\phi_{12},n_{12}\right]$ and ζ for the case of $f_{1}^{*}<P_{D}^{x}$, an auxiliary function $H(f_{1},c)=\Gamma (f_{1})-cf_{1}$ is provided. There exists at least one $c_{0}>0$ that makes $H(f_{1},c)$ obtain the same minimum value marked as υ in two intervals $I_{1}=[0,P_{D}^{x}]$ and $I_{2}=[P_{D}^{x},1]$. The maximum $f_{1}$ corresponding to υ in $I_{1}$ and $I_{2}$ are expressed by $f_{11}(c_{0})$ and $f_{12}(c_{0})$, respectively. As a result, the optimal false-alarm probability $P_{FA,opt}^{y}$ and the corresponding detection probability $P_{D}^{y}$ can be calculated as

(14) $$P_{FA,opt}^{y}=\zeta F_{0,\phi_{11}}(n_{11})+(1-\zeta )F_{0,\phi_{12}}(n_{12})<P_{FA}^{y},$$

(15) $$P_{D}^{y}=\zeta F_{1,\phi_{11}}(n_{11})+(1-\zeta )F_{1,\phi_{12}}(n_{12})=P_{D}^{x},$$

where $\zeta =\left(f_{12}(c_{0})-P_{D}^{x}\right)/\left(f_{12}(c_{0})-f_{11}(c_{0})\right)$, $\phi_{11}$ and $n_{11}$ are determined by $F_{0,\phi_{11}}(n_{11})=\upsilon +c_{0}f_{11}(c_{0})$ and $F_{1,\phi_{11}}(n_{11})=f_{11}(c_{0})$, $\phi_{12}$ and $n_{12}$ are determined by $F_{0,\phi_{12}}(n_{12})=\upsilon +c_{0}f_{12}(c_{0})$ and $F_{1,\phi_{12}}(n_{12})=f_{12}(c_{0})$. As a result, $z_{1}^{o}=P_{FA}^{x}-P_{FA,opt}^{y}$ and $z_{2}^{\left(i\right)}=0$. 

### 4.2. The Optimal Noise Enhanced Solution for Case (ii)

The focus of this subsection is to determine the exact values of the parameters in the optimal noise enhanced solution for case (ii).

Define $G_{\phi }(f_{0})=\sup(f_{1}:F_{0,\phi }(n)=f_{0})$ and $G(f_{0})=\underset{\phi \in \Phi }{\sup}G_{\phi }(f_{0})$, such that $G_{\phi }(f_{0})$ and $G(f_{0})$ are the maximum $f_{1}$ corresponding to a given $f_{0}$ for a fixed ϕ and all ϕ∈Φ, respectively. In addition, $F_{1M}=\max(G(f_{0}))$ since $F_{1M}$ denotes the maximum $f_{1}$, and the minimum $f_{0}$ corresponding to $F_{1M}$ can be denoted by $f_{0}^{*}=\arg\underset{f_{0}}{\min}(G(f_{0})=F_{1M})$. Combined with the location of $f_{0}^{*}$ and Theorem 1, the following theorem is obtained.

**Theorem** **3.** If $f_{0}^{*}\leq P_{FA}^{x}$, then $P_{D,opt}^{y}=F_{1M}>P_{D}^{x}$ and $P_{FA}^{y}=f_{0}^{*}$, the maximum achievable $P_{D}^{y}$ in case (i) is obtained by choosing the detector $\phi_{2o}$ and adding a constant vector $n_{2o}$ to x. Otherwise, the maximization of $P_{D}^{y}$ in case (ii) is obtained by a randomization of two pairs of $\left[\phi_{21},n_{21}\right]$ and $\left[\phi_{22},n_{22}\right]$ with the probabilities λ and 1−λ, respectively, and the corresponding $P_{FA}^{y}=P_{FA}^{x}$. The corresponding proof is similar to that of Theorem 2 and omitted.

According to Theorem 3, when $f_{0}^{*}\leq P_{FA}^{x}$, the detector $\phi_{2o}$ and constant vector $n_{2o}$ that maximizes $P_{D}^{y}$ in case (ii) is determined by $F_{0,\phi_{2o}}(n_{2o})=f_{0}^{*}$ and $F_{1,\phi_{2o}}(n_{2o})=F_{1M}$. Also, $z_{1}^{\left(ii\right)}=P_{FA}^{x}-f_{0}^{*}$ and $z_{2}^{o}=F_{1M}-P_{D}^{x}$.

In addition, in order to determine the exact values of $\left[\phi_{21},n_{21}\right]$, $\left[\phi_{22},n_{22}\right]$ and λ that maximizes $P_{D}^{y}$ when $f_{0}^{*}>P_{FA}^{x}$, we define an auxiliary function that $J(f_{0},k)=G(f_{0})-kf_{0}$. There is at least one $k_{0}>0$ that makes $J(f_{0},k)$ obtain the same maximum value denoted by ν in two intervals $T_{1}=[0,P_{FA}^{x}]$ and $T_{2}=[P_{FA}^{x},1]$. The minimum $f_{0}$ corresponding to ν in $T_{1}$ and $T_{2}$ are expressed by $f_{01}(k_{0})$ and $f_{02}(k_{0})$, respectively. As a consequence, the optimal detection probability $P_{D,opt}^{y}$ and the corresponding false-alarm probability $P_{FA}^{y}$ are recalculated as

(16) $$P_{D,opt}^{y}=\lambda F_{1,\phi_{21}}(n_{21})+(1-\lambda )F_{1,\phi_{22}}(n_{22})>P_{D}^{x},$$

(17) $$P_{FA}^{y}=\lambda F_{0,\phi_{21}}(n_{21})+(1-\lambda )F_{0,\phi_{22}}(n_{22})=P_{FA}^{x},$$

where $\lambda =\left(f_{02}(k_{0})-P_{FA}^{x}\right)/\left(f_{02}(k_{0})-f_{01}(k_{0})\right)$, $\phi_{21}$ and $n_{21}$ are determined by the two equations $F_{0,\phi_{21}}(n_{21})=f_{01}(k_{0})$ and $F_{1,\phi_{21}}(n_{21})=\nu +k_{0}f_{01}(k_{0})$, $\phi_{22}$ and $n_{22}$ are determined by $F_{0,\phi_{22}}(n_{22})=$ $f_{02}(k_{0})$ and $F_{1,\phi_{22}}(n_{22})=\nu +k_{0}f_{02}(k_{0})$. Then we have $z_{1}^{\left(ii\right)}=0$ and $z_{2}^{o}=P_{D,opt}^{y}-P_{D}^{x}$.

### 4.3. The Suitable Noise Solution for Case (iii)

According to the analyses in Section 4.1 and Section 4.2, the model in (9) can be achieved by choosing $\left[\phi_{1o},n_{1o}\right]$ if $f_{1}^{*}>P_{D}^{x}$ and/or choosing $\left[\phi_{2o},n_{2o}\right]$ if $f_{0}^{*}<P_{FA}^{x}$. When $f_{1}^{*}>P_{D}^{x}$ and $f_{0}^{*}<P_{FA}^{x}$ hold at the same time, the model can also be achieved by a randomization of two detectors and noise pairs $\left[\phi_{1o},n_{1o}\right]$ and $\left[\phi_{2o},n_{2o}\right]$ with probabilities η and 1−η, respectively, where 0≤η≤1.

If $f_{1}^{*}\leq P_{D}^{x}$ and $f_{0}^{*}\geq P_{FA}^{x}$, the model in (9), i.e., case (iii) can be achieved by the randomization of the two optimal noise enhanced solutions for case (i) and case (ii) with probabilities η and 1−η, respectively, where 0<η<1. In other words, case (iii) can be achieved by a suitable randomization of $\left[\phi_{11},n_{11}\right]$, $\left[\phi_{12},n_{12}\right]$, $\left[\phi_{21},n_{21}\right]$, and $\left[\phi_{22},n_{22}\right]$ with probabilities ηζ, η(1−ζ), (1−η)λ, and (1−η)(1−λ), respectively, as shown in Table 1.

The corresponding false-alarm and detection probabilities are calculated as

(18) $$P_{FA}^{y}=\eta \zeta F_{0,\phi_{11}}(n_{11})+\eta (1-\zeta )F_{0,\phi_{12}}(n_{12})+(1-\eta )P_{FA}^{x}<P_{FA}^{x},$$

(19) $$P_{D}^{y}=\eta P_{D}^{x}+(1-\eta )\lambda F_{1,\phi_{21}}(n_{21})+(1-\eta )(1-\lambda )F_{1,\phi_{22}}(n_{22})>P_{D}^{x},$$

where 0≤ζ≤1, 0≤λ≤1 and 0<η<1. Especially, η=1 denotes case (i) and η=0 denotes case (ii). It is clearly that different available false-alarm and detection probabilities can be obtained by adjusting the value of η under the constraints that $P_{FA}^{y}\leq P_{FA}^{x}$ and $P_{D}^{y}>P_{D}^{x}$.

From the perspective of Bayesian criterion, the noise modified Bayes risk can be expressed in the form of a false-alarm and detection problem such that

(20) $$R^{\prime }=p(H_{0})C_{00}+p(H_{1})C_{01}+p(H_{0})(C_{10}-C_{00})P_{FA}^{y}-p(H_{1})(C_{01}-C_{11})P_{D}^{y}.$$

where $p(H_{i})$ is the prior probability of $H_{i}$, $C_{ji}$ is the cost of choosing $H_{j}$ when $H_{i}$ is true, i,j=0,1, and $C_{ji}>C_{ii}$ if j≠i. According to case (iii), the improvement ΔR of Bayes risk can be obtained by

(21) $$\Delta R=R-R^{\prime }=p(H_{0})(C_{10}-C_{00})z_{1}+p(H_{1})(C_{01}-C_{11})z_{2}<0,$$

where R is the Bayes risk of the original detector. As a result, case (iii) provides a solution to decrease the Bayes risk.

When $p(H_{i})$ are unknown and $C_{ji}$ are known, an alternative method considers the Minimax criterion, i.e., $\min\{\max({R^{\prime }}_{0},{R^{\prime }}_{1})\}$, where ${R^{\prime }}_{0}=C_{00}+(C_{10}-C_{00})P_{FA}^{y}$ and ${R^{\prime }}_{1}=C_{01}-(C_{01}-C_{11})P_{D}^{y}$ are the conditional risks of choosing $H_{0}$ and $H_{1}$, respectively, for the noise modified detector. Accordingly, case (i) and case (ii) also provide the optimal noise enhanced solution to minimize ${R^{\prime }}_{0}$ and ${R^{\prime }}_{1}$, respectively, for the variable case.

The minimization of Bayes risk for a variable detector has also been discussed in [25]. Compared to this paper, the minimum Bayes risk is obtained without considering the false-alarm and detection probabilities, which is the biggest difference between reference [25] and our work. In addition, the minimization of Bayes risk in [25] is studied only under uniform cost assignment (UCA), i.e., $C_{10}=C_{01}=1$ and $C_{00}=C_{11}=0$, when $p(H_{i})$ is known.

## 5. Numerical Results

In this section, numerical detection examples are given to verify the theoretical conclusions presented in the previous sections. A binary hypothesis-testing problem is given by

(22) $$\left\{ \begin{array}{l} H_{0}:x[i]=\omega [i]\\ H_{1}:x[i]=A+\omega [i] \end{array}\right.,$$

where x is a K-dimensional observation vector, i=0,…,K−1, A>0 is a known signal and ω[i] are i.i.d. symmetric Gaussian mixture noise samples with the pdf

(23) $$p_{\omega }(\omega )=0.5\varphi (\omega ;-\mu ,\sigma^{2})+0.5\varphi (\omega ;\mu ,\sigma^{2}),$$

where $\varphi (\omega ;\mu ,\sigma^{2})=(1/\sqrt{2\pi \sigma^{2}})\exp(-{\left(\omega -\mu \right)}^{2}/2\sigma^{2})$. Let μ=3, A=1, and σ=1. A general decision process of a suboptimal detector is expressed as

(24) $$T(x)\begin{array}{c} H_{1}\\ >\\ <\\ H_{0} \end{array}\gamma ,$$

where γ is the decision threshold.

### 5.1. A Detection Example for K=1

In this subsection, suppose that K=1 and

(25) T(x)=x.

The corresponding decision function is

(26) $$\phi (x)=\left\{ \begin{array}{l} 1,\;{}_{}^{}x\geq \gamma \\ 0,\;x<\gamma \end{array}\right..$$

When we add an additive noise n to x, the noise modified decision function can be written as

(27) $$\phi (y=x+n)=\left\{ \begin{array}{l} 1,\;x+n\geq \gamma \\ 0,\;x+n<\gamma \end{array}\right.=\left\{ \begin{array}{l} 1,\;x+(n-\gamma )\geq 0\\ 0,\;x+(n-\gamma )<0 \end{array}\right.$$

It is obvious from (27) that the detection performance obtained by setting the threshold as γ and adding a noise n is identical with that achieved by keeping the threshold as zero and adding a noise n−γ. As a result, the optimal noise enhanced performances obtained are the same for different thresholds. That is also to say, the randomization between different thresholds cannot improve the optimum performance further and only the non-randomization case should be considered in this example. According to (8), we have

(28) $$F_{0,\phi }(n)=\int_{-\infty }^{\infty }\phi (y)p_{0}(y-n)dn=\frac{1}{2}Q(\frac{\gamma -n-\mu }{\sigma_{0}})+\frac{1}{2}Q(\frac{\gamma -n+\mu }{\sigma_{0}}),$$

(29) $$F_{1,\phi }(n)=\int_{-\infty }^{\infty }\phi (y)p_{1}(y-n)dy=\frac{1}{2}Q(\frac{\gamma -n-\mu -A}{\sigma_{0}})+\frac{1}{2}Q(\frac{\gamma -n+\mu -A}{\sigma_{0}}),$$

where $Q(x)=\int_{x}^{+\infty }\frac{1}{\sqrt{2\pi }}\exp(-\frac{t^{2}}{2})dt$. Based on the analysis on Equation (8), the original false-alarm and detection probabilities are $P_{FA,\phi }^{x}=F_{0,\phi }(0)=\frac{1}{2}Q(\frac{\gamma -\mu }{\sigma_{0}})+\frac{1}{2}Q(\frac{\gamma +\mu }{\sigma_{0}})$ and $P_{D,\phi }^{x}=F_{1,\phi }(0)=\frac{1}{2}Q(\frac{\gamma -\mu -A}{\sigma_{0}})$ $+\frac{1}{2}Q(\frac{\gamma +\mu -A}{\sigma_{0}})$, respectively.

From the definition of function Q, $F_{1,\phi }(n)$, and $F_{0,\phi }(n)$ are monotonically increasing with n and $F_{1,\phi }(n)>F_{0,\phi }(n)$ for any n. In addition, both $F_{1,\phi }(n)$ and $F_{0,\phi }(n)$ are one-to-one mapping functions w.r.t. n. Therefore, we have $\Gamma_{\phi }(f_{1})=F_{0,\phi }(F_{1,\phi }^{-1}(f_{1}))=F_{0,\phi }(n)=f_{0}$ and $G_{\phi }(f_{0})=F_{1,\phi }(F_{0,\phi }^{-1}(f_{0}))=F_{1,\phi }(n)=f_{1}$ for any $\phi^{\prime }$. Furthermore, $\Gamma (f_{1})=\Gamma_{\phi }(f_{1})$ and $G(f_{0})=G_{\phi }(f_{0})$. The relationship between $f_{01,\phi }(k_{0})$ and $f_{11,\phi }(c_{0})$ is one-to-one, as well as that between $f_{02,\phi }(k_{0})$ and $f_{12,\phi }(c_{0})$. As a result, $f_{01,\phi }(k_{0})=f_{01}(k_{0})$, $f_{11,\phi }(c_{0})=f_{11}(c_{0})$, $f_{02,\phi }(k_{0})=f_{02}(k_{0})$, and $f_{12,\phi }(c_{0})=f_{12}(c_{0})$ for any ϕ. That is to say, $n_{11}=n_{21}$ and $n_{12}=n_{22}$, where $\left(n_{11},n_{12}\right)$ and $\left(n_{21},n_{22}\right)$ are the respective optimal noise components for case (i) and case (ii) for any γ. From [2,24], we have

(30) $$n_{11}=n_{21}=\gamma -\mu -0.5A,$$

(31) $$n_{12}=n_{22}=\gamma +\mu +0.5A.$$

**Figure 1:**
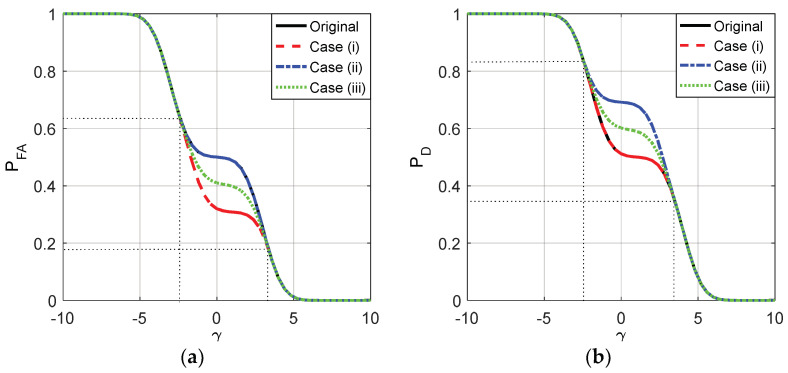
$P_{FA}$ and $P_{D}$ as functions of γ for the original detector and the three cases where μ=3, A=1, and σ=1

Then the pdf of the optimal additive noise corresponding to case (i) and case (ii) for the detector given in (26) can be expressed as

(32) $$p_{1,\phi }^{opt}(n)=\zeta \delta (n-n_{11})+(1-\zeta )\delta (n-n_{12}),$$

(33) $$p_{2,\phi }^{opt}(n)=\lambda \delta (n-n_{21})+(1-\lambda )\delta (n-n_{22}),$$

where $\zeta =\left(F_{1,\phi }(n_{12})-P_{D,\gamma }^{x}\right)/\left(F_{1,\phi }(n_{12})-F_{1,\phi }(n_{11})\right)$ and $\lambda =\left(F_{0,\phi }(n_{22})-P_{FA,\gamma }^{x}\right)/\left(F_{0,\phi }(n_{22})-F_{0,\phi }(n_{21})\right)$. Thus the suitable additive noise for case (iii) can be given by

(34) $$p_{3,\phi }(n)=\eta p_{1,\phi }^{opt}(n)+(1-\eta )p_{2,\phi }^{opt}(n),$$

where 0≤η≤1. In this example, let η=0.5. The false alarm and detection probabilities for the three cases versus different γ are shown in Figure 1.

**Figure 2:**
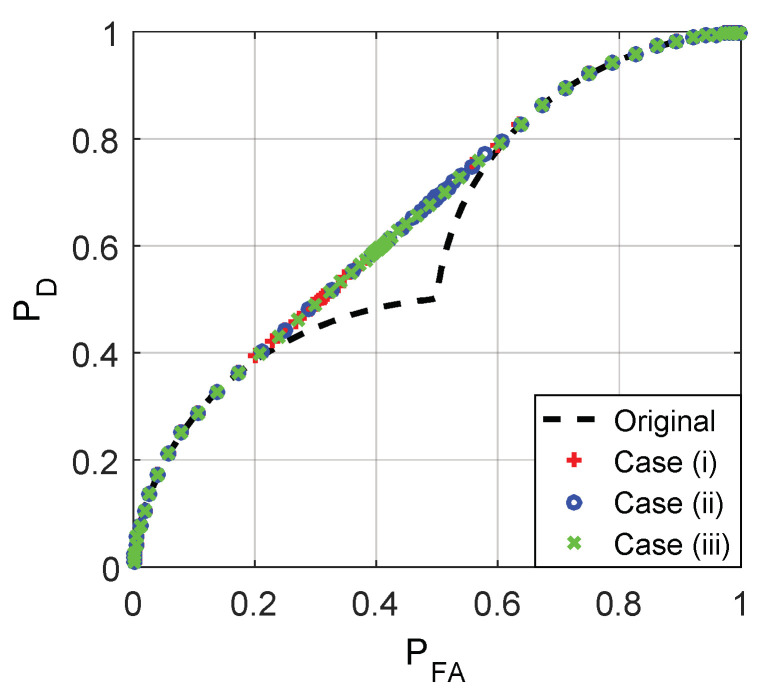
Receiver operating characteristic (ROC) curves for the original detector and the three cases where μ=3, A=1, and σ=1.

As plotted in Figure 1, with the increase of γ, the false alarm and detection probabilities for the three cases and the original detector gradually decrease from 1 to 0, and the noise enhanced phenomenon only occurs when γ∈(−2.5,3.5). Namely, the detection performance can be improved by adding additive noise when the value of γ is between −2.5 and 3.5. When γ∈(−2.5,3.5), $f_{11}(c_{0})<P_{D,\phi }^{x}<f_{12}(c_{0})$ and $f_{01}(k_{0})<P_{FA,\phi }^{x}<f_{02}(k_{0})$, the corresponding 0<ζ<1 and 0<λ<1, thereby the additive noises as shown in (32) and (33) exist to improve the detection performance. Furthermore, the receiver operating characteristic (ROC) curves for the three cases and the original detector are plotted in Figure 2. The ROC curves for the three cases overlap with each other exactly, and the detection probability can be increased by adding additive noise only when the false-alarm probability is between 0.1543 and 0.6543.

Given $C_{ji}$ and the prior probability $p(H_{i})$, i,j=0,1, the noise enhanced Bayes risk obtained according to case (iii) is given by

(35) $$R_{iii,,\phi }=R-[p(H_{0})(C_{10}-C_{00})\eta z_{1,\phi }^{o}+p(H_{1})(C_{01}-C_{11})(1-\eta )z_{2,\phi }^{o}],$$

where $z_{1,\phi }^{o}=P_{FA,\phi }^{x}-\int p_{3,\phi }(n)F_{0,\phi }(n)dn$ and $z_{2,\phi }^{o}=\int p_{3,\phi }(n)F_{1,\phi }(n)dn-P_{D,\phi }^{x}$. Let η=0.5, $C_{10}=C_{01}=1$ and $C_{00}=C_{11}=0$, then the Bayes risk of the original detector is calculated as

(36) $$R=p(H_{0})P_{FA}^{x}-p(H_{1})(1-P_{D}^{x}).$$

**Figure 3:**
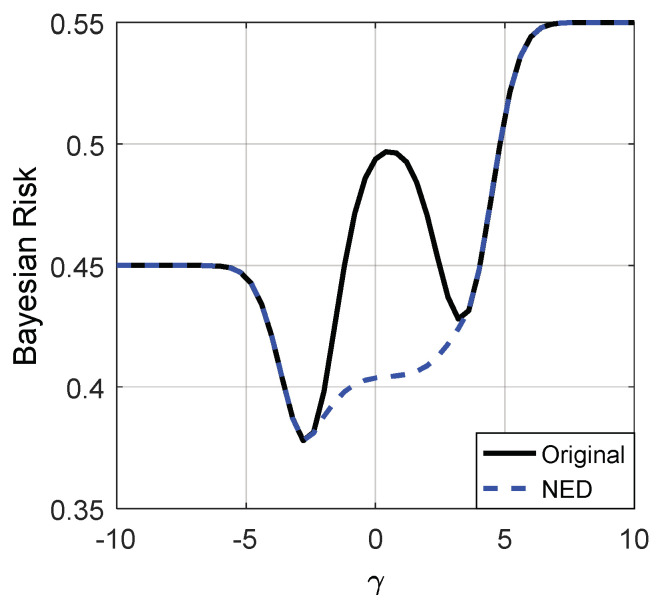
Bayes risks of the noise enhanced and original detectors for $p(H_{0})=0.45$. “NED” denotes the noise enhanced detector.

**Figure 4:**
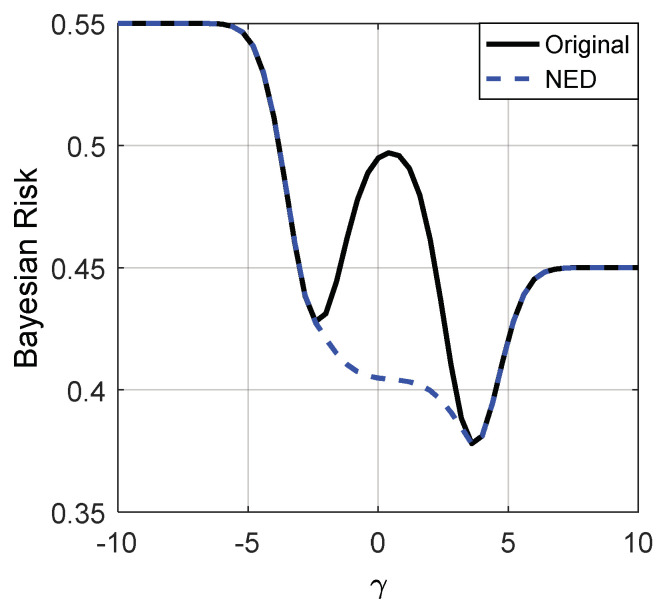
Bayes risks of the noise enhanced and the original detectors for $p(H_{0})=0.55$.

Figure 3 and Figure 4 depict the Bayes risks of the noise enhanced and the original detectors versus different γ for $p(H_{0})=0.45$ and 0.55, respectively.

From Figure 3 and Figure 4, we can see that when the decision threshold γ is very small, the Bayes risks of the noise enhanced and the original detectors are close to $p(H_{0})$. As illustrated in Figure 3 and Figure 4, only when γ∈(−2.5,3.5), can the Bayes risk be decreased by adding additive noise. With the increase of γ, the difference between the Bayes risks of the noise enhanced detector and the original detector first increases and then decreases to zero, and reaches the maximum value when γ=0.5. If the decision threshold γ is large enough, the Bayes risks for the two detectors are close to $p(H_{1})=1-p(H_{0})$. In addition, there is no link between the values of $p(H_{0})$ and the possibility of the detection performance can or cannot be improved via additive noise, which is consistent with (35).

### 5.2. A Detection Example for K=2

In this example, suppose that

(37) $$T(x)=\frac{1}{K}\sum_{i=0}^{K-1}S(x[i]),$$

where $S(x[i])=\left\{ \begin{array}{l} 1,x[i]\geq 0\\ 0,x[i]<0 \end{array}\right.$. When K=2, we have

(38) $$T(x)=\frac{1}{2}\sum_{i=0}^{1}\left(S(x[i])\right)=\left\{ \begin{array}{l} 1,\;S(x[0])\geq 0\;and\;S(x[1])\geq 0\\ 0.5,\;S(x[0])\geq 0\;or\;S(x[1])\geq 0\\ 0,\;S(x[0])<0\;and\;S(x[1])<0 \end{array}\right..$$

It is obvious that T(x)>γ when γ<0 and T(x)<γ when γ>1, which implies the detection result is invalid if γ<0 or γ>1. In addition, the detection performance is the same for γ∈(0,0.5] (γ∈(0.5,1]). Therefore, suppose that two alternative thresholds are γ=1 and γ=0.5, the corresponding decision functions are denoted by $\phi_{1}$ and $\phi_{2}$, respectively. Let $n={\left(n_{1},n_{2}\right)}^{T}$, then we have

(39) $$F_{i,\phi_{1}}(n)=F_{i,\phi }(n_{1})F_{i,\phi }(n_{2}),$$

(40) $$F_{i,\phi_{2}}(n)=1-(1-F_{i,\phi }(n_{1}))(1-F_{i,\phi }(n_{2})),$$

where i=0,1, ϕ(⋅) is the decision function given in (26) with γ=0. Based on the theoretical analysis in Section 4, $\Gamma_{\phi_{i}}(f_{1})=\min(F_{0,\phi_{i}}(n):F_{1,\phi_{i}}(n)=f_{1})$, and $G_{\phi_{i}}(f_{0})=\max(F_{1,\phi_{i}}(n):F_{0,\phi_{i}}(n)=f_{0})$. Furthermore, $\Gamma (f_{1})=\min(\Gamma_{\phi_{1}}(f_{1}),\Gamma_{\phi_{2}}(f_{1}))$ and $G(f_{0})=\max(G_{\phi_{1}}(f_{0}),G_{\phi_{2}}(f_{0}))$.

Through a series of analyses and calculations, it is true that $\Gamma_{\phi_{1}}(f_{1})=\min(F_{0,\phi }^{2}(n_{1}),F_{0,\phi }(n_{2}))$, where $n_{1}$ and $n_{2}$ are determined by $F_{1,\phi }^{2}(n_{1})=f_{1}$ and $F_{1,\phi }(n_{2})=f_{1}$, respectively. Similarly, $\Gamma_{\phi_{2}}(f_{1})=\min(1-{\left(1-F_{0,\phi }(n_{1})\right)}^{2},1-(1-F_{0,\phi }(n_{2})))$, where $n_{1}$ and $n_{2}$ are determined by $1-{\left(1-F_{1,\phi }(n_{1})\right)}^{2}=f_{1}$ and $1-(1-F_{1,\phi }(n_{2}))=f_{1}$, respectively. Moreover, $\Gamma (f_{1})=\min(F_{0,\phi }^{2}(n_{1}),1-{\left(1-F_{0,\phi }(n_{2})\right)}^{2})$ where $F_{1,\phi }^{2}(n_{1})=1-{\left(1-F_{1,\phi }(n_{2})\right)}^{2}=f_{1}$.

On the other hand, $G_{\phi_{1}}(f_{0})=\max(F_{1,\phi }^{2}(n_{1}),F_{1,\phi }(n_{2}))$ where $n_{1}$ and $n_{2}$ are determined by $F_{0,\phi }^{2}(n_{1})=f_{0}$ and $F_{0,\phi }(n_{2})=f_{0}$. Moreover, $G_{\phi_{2}}(f_{0})=\max(1-{\left(1-F_{1,\phi }(n_{1})\right)}^{2},1-(1-F_{1,\phi }(n_{2})))$, where $1-{\left(1-F_{0,\phi }(n_{1})\right)}^{2}=f_{0}$ and $1-(1-F_{0,\phi }(n_{2}))=f_{0}$. As a consequence, $G(f_{0})=\max(F_{1,\phi }^{2}(n_{1}),1-{\left(1-F_{1,\phi }(n_{2})\right)}^{2})$ where $F_{0,\phi }^{2}(n_{1})=1-{\left(1-F_{0,\phi }(n_{2})\right)}^{2}=f_{0}$.

**Figure 5:**
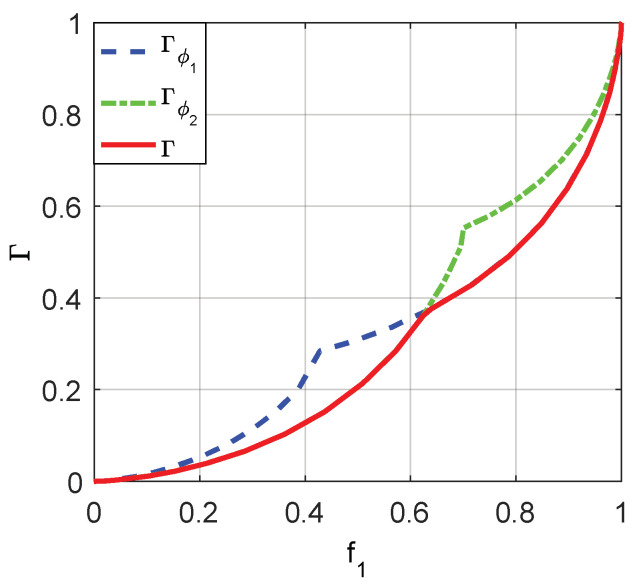
The relationships between $\Gamma_{\phi_{1}}(f_{1})$, $\Gamma_{\phi_{2}}(f_{1})$ and $\Gamma (f_{1})$.

**Figure 6:**
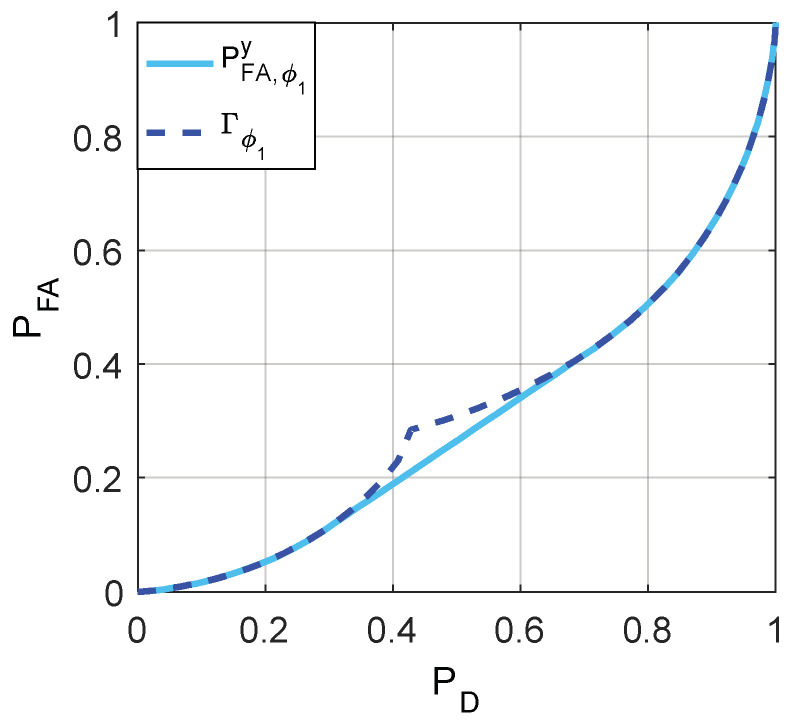
$\Gamma_{\phi_{1}}(f_{1})$ and the achievable minimum $P_{FA}$ obtained when γ=1.

**Figure 7:**
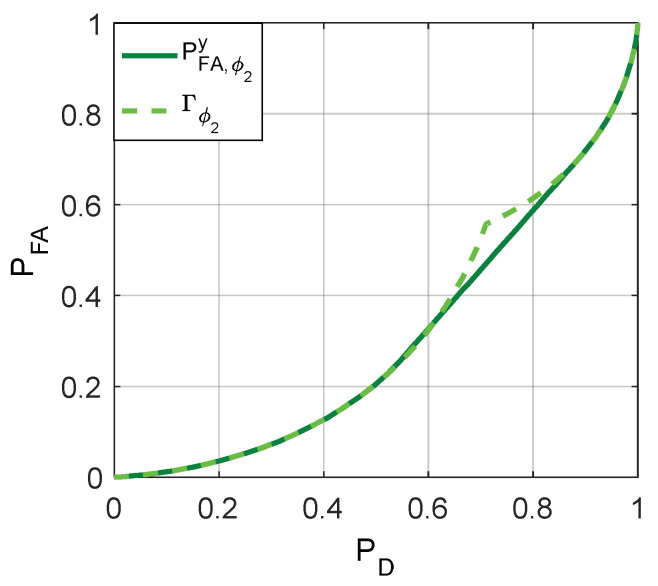
The achievable minimum $P_{FA}$ obtained when γ=0.5 and $\Gamma_{\phi_{2}}(f_{1})$.

**Figure 8:**
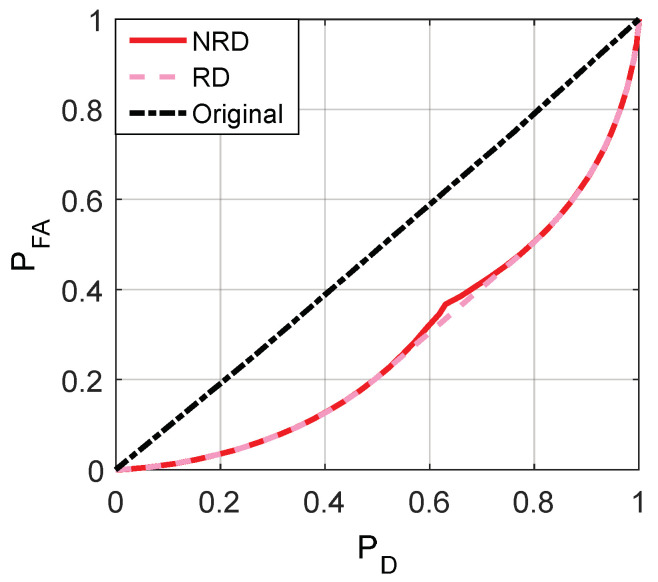
Comparison of minimum $P_{FA}$ achieved by the original detector and noise enhanced decisions for “NRD” and “RD” cases, which denote non-randomization and randomization exist between the thresholds, respectively.

The minimum achievable false-alarm probabilities for $\phi_{1}$ and $\phi_{2}$, i.e., $P_{FA,\phi_{1}}^{y}$ and $P_{FA,\phi_{2}}^{y}$, can be obtained, respectively, by utilizing the relationships between $\Gamma_{\phi_{1}}(f_{1})$, $\Gamma_{\phi_{2}}(f_{1})$, and $\Gamma (f_{1})$ as depicted in Figure 5. Then Figure 6, Figure 7 and Figure 8 are given to illustrate the relationship between $P_{FA}$ and $P_{D}$ clearly under two different decision thresholds. As illustrated in Figure 6, for the case of threshold γ=1, the false-alarm probability can be decreased by adding an additive noise when $0.2994\leq P_{D}\leq 0.7153$. Correspondingly, the false-alarm probability can be decreased by adding an additive noise only when $0.5416\leq P_{D}\leq 0.8867$ for γ=0.5 as shown in Figure 7. The minimum false-alarm probability for a given $P_{D}$ without threshold randomization is $P_{FA,m}^{y}=\min(P_{FA,\phi_{1}}^{y},P_{FA,\phi_{2}}^{y})$, which is plotted in Figure 8 and represented by the legend “NRD”.

As illustrated in Figure 8, when the randomization between decision thresholds is allowed, the noise modified false-alarm probability can be decreased further compared with the case where no randomization between decision thresholds is allowed for $0.5091\leq P_{D}\leq 0.7862$. Actually, the minimum achievable noise modified false-alarm probability is obtained by a suitable randomization between two threshold and discrete vector pairs, i.e., $\left\{\gamma =0.5,n_{11}=[-3.75,-3.75]\right\}$ and $\left\{\gamma =1,\;n_{12}=[2.75,2.75]\right\}$, with probabilities ζ and 1−ζ, respectively, such that

(41) $$\zeta =\frac{0.7862-P_{D}}{0.2771}.$$

Remarkably, the minimum false-alarm probability obtained in the “NRD” case is always superior to the original false-alarm probability for any $P_{D}$.

If the randomization between different decision thresholds is not allowed, the detection probability can be increased by adding additive noise when $0.1133\leq P_{FA}\leq 0.4281$ and $0.2501\leq P_{FA}\leq 0.7006$ for γ=1 and γ=0.5, respectively. When the randomization is allowed, for $0.2164\leq P_{FA}\leq 0.4909$, the maximum achievable detection probability can be obtained by a randomization of two pairs $\left\{\gamma =0.5,n_{21}=[-3.75,-3.75]\right\}$ and $\left\{\gamma =1,n_{22}=[2.75,2.75]\right\}$ with the corresponding weights $\lambda =\frac{0.4909-P_{FA}}{0.2745}$ and 1−λ.

**Figure 9:**
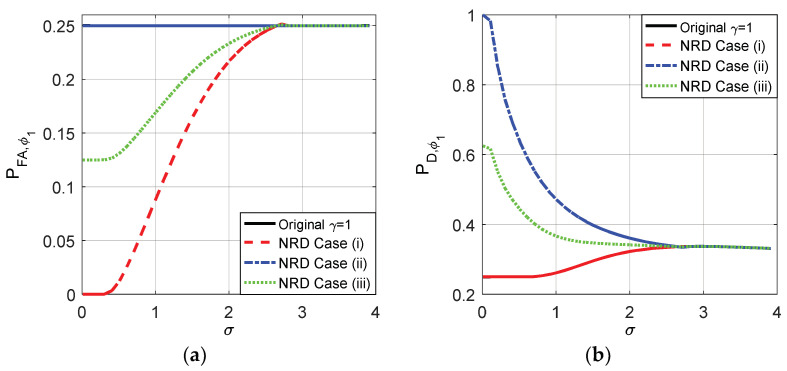
$P_{FA}$ and $P_{D}$ as functions of σ for the original detector and the three cases without randomization existing between thresholds when γ=1 for μ=3, A=1, and η=0.5.

**Figure 10:**
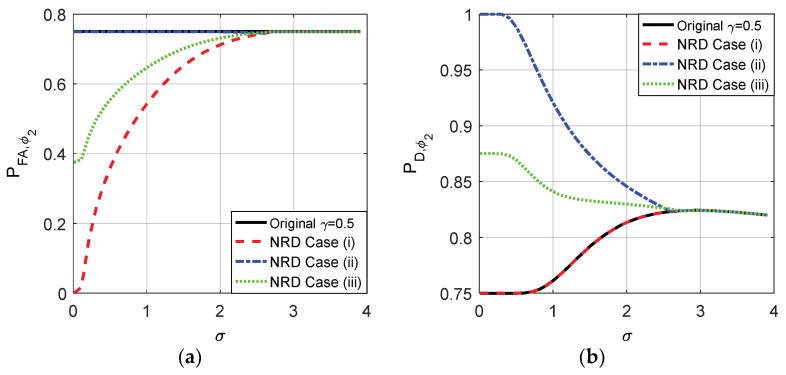
$P_{FA}$ and $P_{D}$ as functions of σ for the original detector and the three cases without randomization exisingt between thresholds when γ=0.5 for μ=3, A=1, and η=0.5.

The probabilities of false-alarm and detection for different σ of the original detector and cases (i), (ii), and (iii) when the decision threshold γ=1 and γ=0.5 are compared in Figure 9 and Figure 10, respectively. As shown in Figure 9a,b, the original $P_{FA}$ maintains 0.25 for any σ and the original $P_{D}$ is between 0.25 and 0.3371 when γ=1. As plotted in Figure 10a,b, the original $P_{FA}$ maintains 0.75 and the original $P_{D}$ is between 0.75 and 0.8242 when γ=0.5.

According to the analyses above, the original $P_{FA}$ and $P_{D}$ obtained when γ=1 and γ=0.5 are in the interval where the noise enhanced detection could occur. When the randomization between the thresholds is not allowed, according to the theoretical analysis, the optimal solutions of the noise enhanced detection performance for both case (i) and (ii) are to choose a suitable threshold and add the corresponding optimal noise to the observation. After some comparisons, the suitable threshold is just the original detector under the constraints in which $P_{FA}^{y}\leq P_{FA}^{x}$ and $P_{D}^{y}\geq P_{D}^{x}$ in this example. Naturally, case (iii) can be achieved by choosing the original detector and adding the noise which is a randomization between two optimal additive noises obtained in case (i) and case (ii). The details are plotted in Figure 9 and Figure 10 for the original threshold γ=1 and 0.5, respectively.

From Figure 9 and Figure 10, it is clear that the smaller the σ, the smaller the false-alarm probability and the larger the detection probability. When σ is close to 0, the false-alarm probability obtained in case (i) is close to 0 and the detection probability obtained in case (ii) is close to 1. As shown in Figure 9, compared to the original detector, the noise enhanced false-alarm probability and the detection probability obtained in case (iii) are decreased by 0.125 and increased by 0.35, respectively, when σ is close to 0 where γ=1 and η=0.5. As shown in Figure 10, compared to the original detector, the noise enhanced false-alarm probability obtained in case (iii) is decreased by 0.375 and the corresponding detection probability is increased by 0.125, respectively, when γ=0.5 and η=0.5. With the increase of σ, the improvements of false-alarm and detection probabilities decrease gradually to zero as shown in Figure 9 and Figure 10. When σ>2.81, because the pdf of $p_{\omega }(\omega )$ gradually becomes a unimodal noise, the detection performance cannot be enhanced by adding any noise.

**Figure 11:**
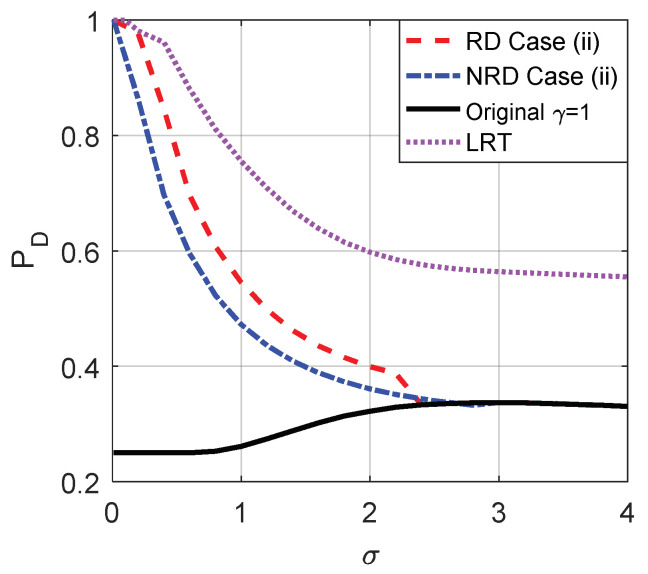
Comparison of $P_{D}$ as function of σ for the original detector, LRT and case (ii) with or without randomization between thresholds, respectively, when μ=3, A=1 and the original threshold γ=1. “LRT” is the $P_{D}^{x}$ obtained under the Likelihood ratio test (LRT) based on the original observation x.

After some calculations, we know that under the constraints that $P_{FA}^{y}\leq P_{FA}^{x}$ and $P_{D}^{y}\geq P_{D}^{x}$, the false-alarm probability cannot be decreased further by allowing randomization between the two thresholds compared to the non-randomization case when the original threshold is γ=1. It means that even if randomization is allowed, the minimum false-alarm probability in case (i) is obtained by choosing threshold γ=1 and adding the corresponding optimal additive noise, and the achievable minimum false-alarm probability in case (i) is the same as that plotted in Figure 9. On the contrary, the detection probability obtained under the same constraints when randomization exists between different thresholds is greater than that obtained in the non-randomization case for σ<2.3, which is shown in Figure 11. Based on the analysis in Section 3.2, the maximum detection probability in case (ii) can be achieved by a suitable randomization of the two decision thresholds and noise pairs $\left\{\gamma =0.5,n_{21}=[n_{21},n_{21}]\right\}$ and $\left\{\gamma =1,n_{22}=[n_{22},n_{22}]\right\}$ with probabilities λ and 1−λ, respectively, where $\lambda =\left(F_{0,\phi_{1}}(n_{22})-F_{0,\phi_{1}}(0)\right)/\left(F_{0,\phi_{1}}(n_{22})-F_{0,\phi_{2}}(n_{21})\right)$. Such as $n_{21}=[-4.25,-4.25]$, $n_{22}=$ [2.15,2.15], and λ=0.8966 when σ=1.8. In addition, Figure 11 also plots the $P_{D}^{x}$ under the Likelihood ratio test (LRT) based on the original observation x. It is obvious that the $P_{D}$ obtained under LRT is superior to that obtained in case (ii) for each σ. Although the performance of LRT is much better than the original and noise enhanced decision solutions, its implementation is much more complicated.

**Figure 12:**
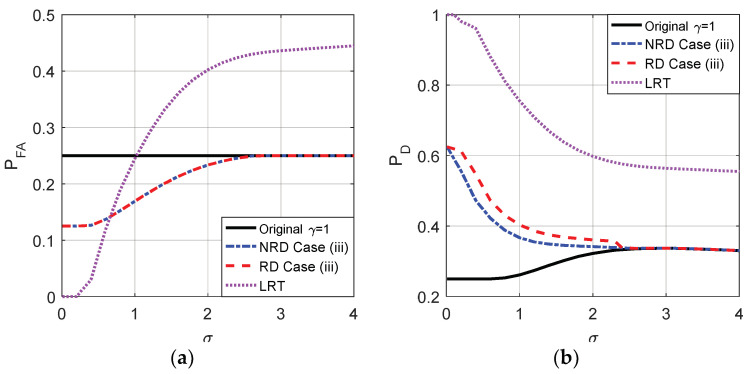
Comparison of $P_{FA}$ and $P_{D}$ as functions of σ for the original detector, LRT and case (iii) with or without randomization between thresholds, respectively, when μ=3, A=1, and the original threshold γ=1.

Naturally, case (iii) can also be achieved by randomization of the noise enhanced solution for case (i) and the new solution for case (ii) with the probabilities η and 1−η, respectively. Figure 12 compares the probabilities of false-alarm and detection obtained by the original detector, LRT and case (iii) when randomization can or cannot be allowed where η=0.5 and the original threshold is γ=1. As shown in Figure 12, compared to the non-randomization case, the detection probability obtained in case (iii) is further improved for σ<2.3 by allowing randomization of the two thresholds while the false-alarm probability cannot be further decreased. Moreover, the $P_{FA}$ of LRT increases when σ increases and will be greater than that obtained in case (iii) when σ>0.66 and the original detector when σ>1.

**Figure 13:**
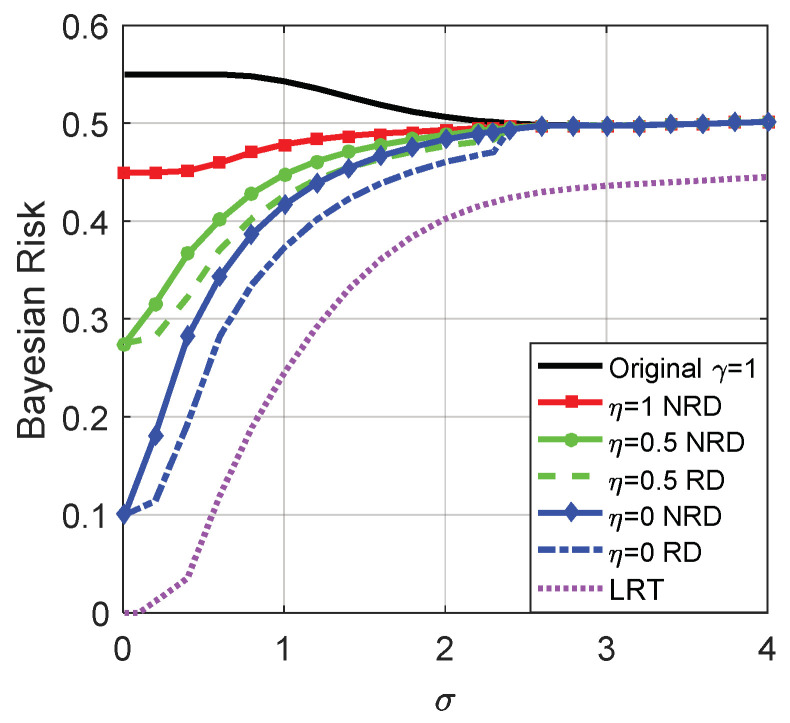
Bayes risks of the original detector, LRT and noise enhanced decision solutions for different η when μ=3, A=1, and the original threshold γ=1.

Figure 13 illustrates the Bayes risks for the original detector, LRT and noise enhanced decision solutions when the randomization between detectors can or cannot be allowed for different η, where η=1, η=0, and η=0.5 denote case (i), case (ii), and case (iii), respectively. As plotted in Figure 13, the Bayes risks obtained in case (i), (ii), and (iii) are smaller than the original detector, and the Bayes risk of LRT is the smallest one. Furthermore, the Bayes risk obtained in the randomization case is smaller than that obtained in the non-randomization case.

**Figure 14:**
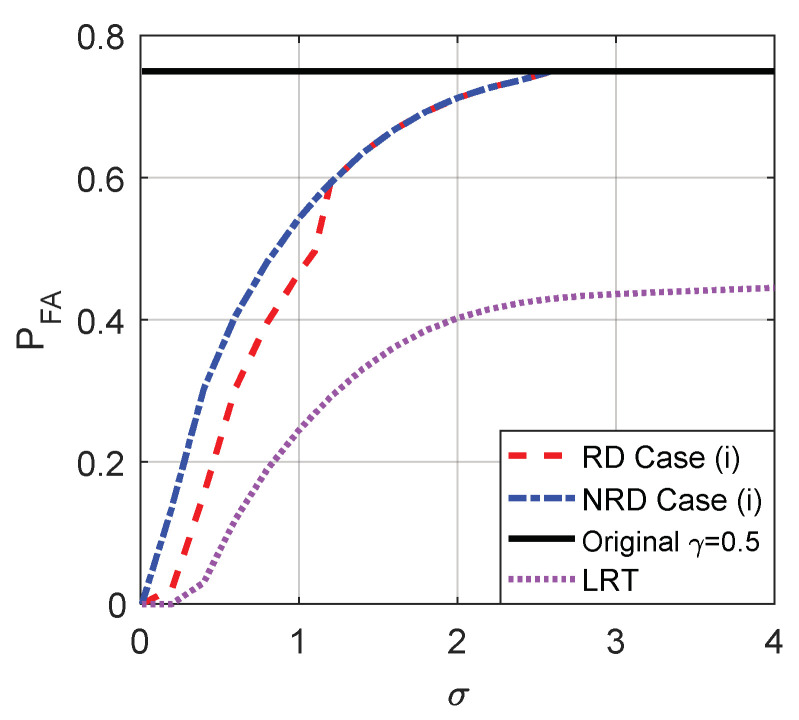
Comparison of $P_{FA}$ as function of σ for the original detector, LRT and case (i) with or without randomization between thresholds, respectively, when μ=3, A=1, and the original threshold γ=0.5.

As shown in Figure 14, when the original threshold γ=0.5, under the constraints that $P_{FA}^{y}\leq P_{FA}^{x}$ and $P_{D}^{y}\geq P_{D}^{x}$, the false-alarm probability can be greatly decreased by allowing randomization between different thresholds compared to the non-randomization case when σ<1.1. In addition, LRT performs best on $P_{FA}$. Accordingly, the minimum false-alarm probability in case (i) is obtained by a suitable randomization of $\left\{\gamma =0.5,n_{21}=[n_{21},n_{21}]\right\}$ and $\left\{\gamma =1,n_{22}=[n_{21},n_{22}]\right\}$ with probabilities ζ and 1−ζ, respectively, where $\zeta =\left(F_{1,\phi_{1}}(n_{22})-F_{1,\phi_{1}}(0)\right)/\left(F_{1,\phi_{1}}(n_{22})-F_{1,\phi_{2}}(n_{21})\right)$. Through some simple analyses, under the same constraints, the detection probability obtained when there exists randomization between different thresholds cannot be greater than that obtained in the non-randomization case. Thus the maximum detection probability in case (ii) is the same as that illustrated in Figure 10, which is achieved by choosing a threshold γ=0.5 and adding the corresponding optimal additive noise to the observation.

**Figure 15:**
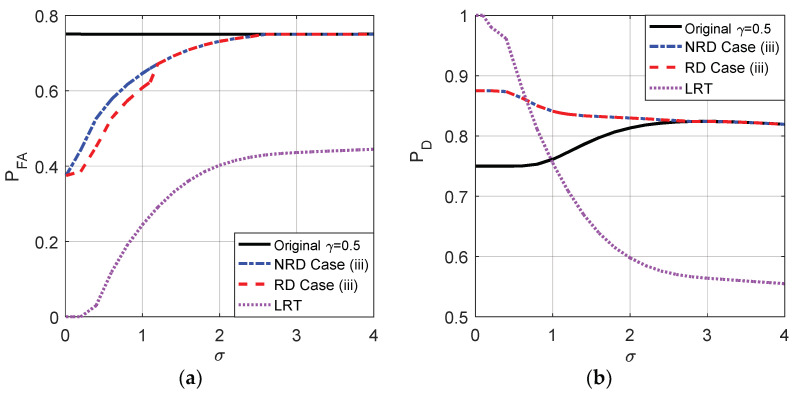
Comparison of $P_{FA}$ and $P_{D}$ as functions of σ for the original detector, LRT and case (iii) with or without randomization between thresholds, respectively, when μ=3, A=1, and the original threshold γ=0.5.

Case (iii) can also be achieved by the randomization of the noise enhanced solutions for case (i) and case (ii) with the probabilities η and 1−η, respectively. As shown in Figure 15, compared to the non-randomization case, the false-alarm probability obtained in case (iii) is greatly improved by allowing randomization of the two thresholds while the detection probability cannot be increased when the original threshold γ=0.5. Although the $P_{FA}$ of LRT is always superior to that obtained in other cases, the $P_{D}$ of LRT will be smaller than that obtained by the original detector and case (iii) when σ increases to a certain extent.

**Figure 16:**
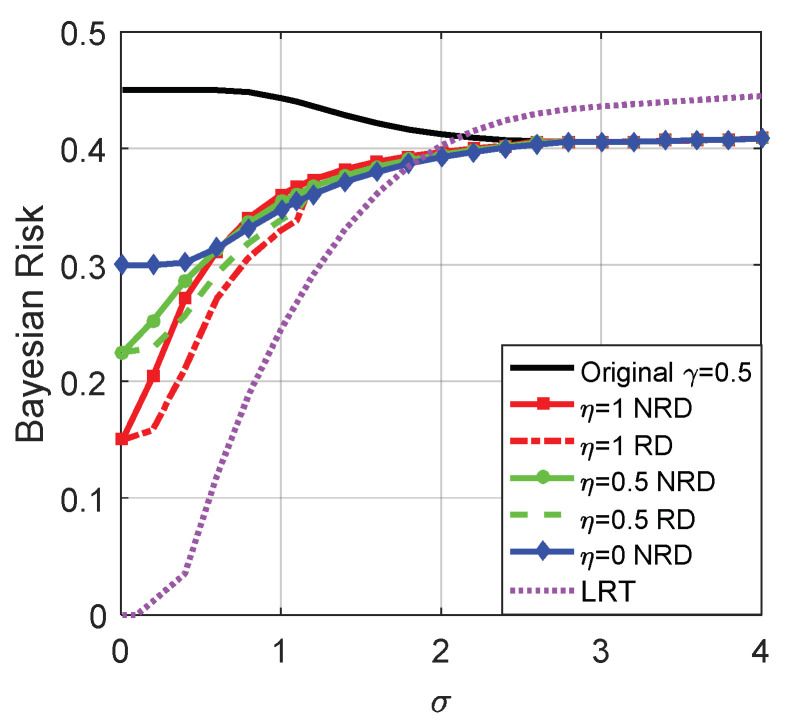
Bayes risks of the original detector, LRT and the noise enhanced decision solutions for different η when μ=3, A=1, and the original threshold γ=0.5.

Figure 16 illustrates the Bayes risks for the original detector, LRT and the noise enhanced decision solutions for different η. Also, η=1, η=0, and η=0.5 denote case (i), case (ii) and case (iii), respectively. As plotted in Figure 16, the Bayes risks obtained by the three cases are smaller than the original detector for σ<2.81. The smallest one of the three is achieved in case (i) if σ<0.7 or case (ii) if 0.7<σ<2.81 when no randomization exists between the thresholds, while it is achieved in case (i) if σ<1.1 or case (ii) if 1.1<σ<2.81 when the randomization between the thresholds is allowed. Obviously, the Bayes risk obtained in the randomization case is not greater than that obtained in the non-randomization case. In addition, LRT achieves the minimum Bayes risk when σ<1.83 and the maximum Bayes risk when σ>2.12.

As analyzed in 5.1, if the structure of a detector does not change with the decision thresholds, the optimal noise enhanced detection performances for different thresholds are the same, which can be achieved by adding the corresponding optimal noise. In such case, no improvement can be obtained by allowing randomization between different decision thresholds. On the other hand, if different thresholds correspond to different structures as shown in (39) and (40), randomization between different decision thresholds can introduce new noise enhanced solutions to improve the detection performance further under certain conditions.

## 6. Conclusions

In this study, a noise enhanced binary hypothesis-testing problem for a variable detector was investigated. Specifically, a noise enhanced model that can increase the detection probability and decrease the false-alarm probability simultaneously was formulated for a variable detector. In order to solve the model, three alternative cases were considered, i.e., cases (i), (ii), and (iii). First, the minimization of the false-alarm probability was achieved without decreasing the detection probability in case (i). For the case where the randomization between different detectors is allowed, the optimal noise enhanced solution of case (i) was proven as a randomization of at most two detectors and additive noise pairs. Especially, no improvement could be introduced by allowing the randomization between different detectors under certain conditions. Furthermore, the maximum noise enhanced detection probability was considered in case (ii) without increasing the false-alarm probability, and the corresponding optimal noise enhanced solution was explored regardless of whether the randomization of detectors is allowed. In addition, case (iii) was achieved by the randomization between two optimal noise enhanced solutions of cases (i) and (ii) with the corresponding weights. Remarkably, numerous solutions for increasing the detection probability and decreasing the false-alarm probability simultaneously are provided by adjusting the weights.

Moreover, the minimization of the Bayes risk based on the noise enhanced model was discussed for the variable detector. The Bayes risk obtained in case (iii) is between that obtained in case (i) and case (ii). Obviously, the noise enhanced Bayes risks obtained in the three cases are smaller than the original one without additive noise. It is significant to investigate case (i) and case (iii), especially for the situation where the result of case (ii) is not ideal or even not exists. Such as shown in Figure 16, the Bayes risks obtained in cases (i) and (iii) are smaller than that obtained in case (ii) under certain conditions. Studies on cases (i) and (iii) further supplement case (ii), and provide a more common noise enhanced solution for the signal detection. Finally, through the simulation results, the theoretical analyses were proven.

As a future work, the noise enhanced detection problem can be researched according to the maximum likelihood (ML) or the maximum a posteriori probability (MAP) criterion. Also, the theoretical results can be extended to the Rao test, which is a simpler alternative to the generalized likelihood ratio test (GLRT) [29], and applied to a stable system, a spectrum sensing problem in cognitive radio systems, and a decentralized detection problem [30,31,32]. In addition, a generalized noise enhanced parameter estimation problem based on the minimum mean square error (MMSE) criterion is another issue worth studying.

## Figures and Tables

**Table 1:** The probability of each component in the suitable noise enhanced solution for case (iii).

[ϕ,n]	$\left[\phi_{11},n_{11}\right]$	$\left[\phi_{12},n_{12}\right]$	$\left[\phi_{21},n_{21}\right]$	$\left[\phi_{22},n_{22}\right]$
probability	ηζ	η(1−ζ)	(1−η)λ	(1−η)(1−λ)

## Appendix A

**Proof** **of** **Theorem 1.** Due to A, U is a two-dimensional linear space. Further, suppose that B is the convex hull of U. It can be testified that B is also the set of all possible $\left(P_{FA}^{y},P_{D}^{y}\right)$. Generally, the optimal pairs of $\left(P_{FA}^{y},P_{D}^{y}\right)$ for case (i) and (ii) can only exist on ℂ, i.e., the boundary of B. Furthermore, according to the Caratheodory’s Theorem, an arbitrary point on ℂ can be denoted by a convex combination of no more than two elements in U. Consequently, case (i) and case (ii) can be achieved by a randomization of at most two detectors and discrete vector pairs.☐

## Appendix B

**Proof** **of** **Theorem 2.** If $f_{1}^{*}\geq P_{D}^{x}$, there must exist a detector $\phi_{1o}$ and a constant vector $n_{1o}$ must satisfy $P_{FA,opt}^{y}=F_{0,\phi_{1o}}(n_{1o})=F_{0m}<P_{FA}^{y}$ and $P_{D}^{y}=F_{1,\phi_{1o}}(n_{1o})=f_{1}^{*}\geq P_{D}^{x}$. Thus, the minimum achievable $P_{FA}^{y}$ is obtained by choosing the detector $\phi_{1o}$ and adding a discrete vector $n_{1o}$ to x. For the case of $f_{1}^{*}<P_{D}^{x}$, the optimal noise enhanced solution that minimizes $P_{FA}^{y}$ is a randomization of two detectors and discrete vector pairs, i.e., $\left[\phi_{11},n_{11}\right]$ and $\left[\phi_{12},n_{12}\right]$ with probabilities ζ and 1−ζ directly according to Theorem 1. In addition, the contradiction method can be used here to prove $P_{D}^{y}=P_{D}^{x}$. First, suppose the minimum false-alarm probability $P_{FA,opt}^{y}$ is obtained when $P_{D,opt}^{y}=d<P_{D}^{x}$ with a noise enhanced solution ${\left\{E[\phi ,n]\right\}}^{opt}$. Then we suppose another valid solution which is a randomization of ${\left\{E[\phi ,n]\right\}}^{opt}$ and $\left[\phi_{1o},n_{1o}\right]$ with probabilities $t=\left(P_{D}^{x}-d\right)/\left(f_{1}^{*}-d\right)$ and 1−t. Then the new noise modified detection and false-alarm probabilities can be calculated as

(42) $$P_{D}^{y}=\frac{P_{D}^{x}-d}{f_{1}^{*}-d}f_{1}^{*}+\frac{f_{1}^{*}-P_{D}^{x}}{f_{1}^{*}-d}d=P_{D}^{x},$$

(43) $$P_{FA}^{y}=\frac{P_{D}^{x}-d}{f_{1}^{*}-d}F_{0m}+\frac{f_{1}^{*}-P_{D}^{x}}{f_{1}^{*}-d}P_{FA,opt}^{y}<P_{FA,opt}^{y},$$

where the last inequality holds from $F_{0m}<P_{FA,opt}^{y}$. Therefore, $P_{D,opt}^{y}=P_{D}^{x}$ since the result in (A2) contracts the definition of $P_{FA,opt}^{y}$.☐
